# Clinical efficacy analysis of elastic fixation for isolated ligamentous Lisfranc injuries

**DOI:** 10.1186/s12893-025-03377-8

**Published:** 2025-12-05

**Authors:** Yang Liu, Hao Wan, Wen Lu, Xiao-lin Ding

**Affiliations:** Department of Orthopedics, The Second Affliated Hospital of Shandong First Medical University, Taian, China

**Keywords:** Lisfranc injury elastic fixation plantar pressure analysis

## Abstract

**Background:**

Elastic fixation represents a novel approach to managing Lisfranc injuries. This study aims to evaluate the clinical efficacy of elastic fixation for such injuries using imaging, clinical evaluation indices, and plantar pressure analysis.

**Methods:**

This retrospective study included 15 patients with isolated ligamentous Lisfranc injuries who underwent elastic fixation between October 2022 and July 2024.Follow-up lasted ≥ 6 months. Weight-bearing foot X-rays were taken at 1, 3, and 6 months postoperatively to assess joint reduction, degeneration, and implant stability. American Orthopaedic Foot and Ankle Society (AOFAS) scores and Foot Function Index (FFI) were collected preoperatively and at 1–6 months postoperatively. Plantar pressure distribution was also measured.

**Results:**

Imaging showed stable reduction of the tarsometatarsal joints without degeneration or implant failure in all patients. Clinical scores improved significantly: AOFAS scores from 37.6 ± 6.75 to 92.9 ± 0.96 (*P* < 0.0001), and FFI from 151.7 ± 9.31 to 10.1 ± 2.59 (*P* < 0.0001) by 6 months postoperatively. Plantar pressure analysis revealed the affected foot’s pressure contribution increased from 20.4% preoperatively to 50.4% at 6 months postoperatively. By 2 months postoperatively, pressure distribution between both feet was balanced (*P* > 0.05).

**Conclusions:**

In this highly selected cohort, elastic fixation achieved excellent short-term functional recovery and allowed early weight-bearing. Owing to the small sample and short follow-up, these findings are exploratory and hypothesis-generating rather than definitive. Larger, longer-term studies are required to validate the safety and generalisability of the technique across broader patient populations.

## Introduction

 Lisfranc injury, or tarsometatarsal joint complex injury, is a rare yet severe midfoot trauma, accounting for approximately 0.2% of all fractures. It predominates in men, with a male-to-female ratio of approximately 4.25:1 [1, 2]. This injury can be purely ligamentous or involve fractures and joint structures [3, 4]. Low-energy trauma typically results in isolated ligamentous damage, which, if improperly treated, may lead to complications like malunion, chronic pain, and midfoot arthritis [5, 6].

Clinically, common internal fixation methods include screw fixation, plate fixation, combined screw-plate fixation, and elastic fixation. However, traditional screw and plate fixations have notable drawbacks. These methods are highly invasive and carry risks of poor wound healing and infection [7]. Moreover, internal fixation devices can damage joints, increasing the risk of post-traumatic arthritis, and usually require a second surgery for removal, prolonging recovery time [8].

In contrast, elastic fixation, a less rigid surgical technique, has gained attention in recent years. It uses elastic materials to reconstruct the Lisfranc ligament, allowing controlled joint micromotion. This not only reduces hardware-related issues from rigid fixation but also provides good biomechanical stability for the foot, aiding joint function recovery. Despite its advantages, past evaluations of elastic fixation’s postoperative effects have been relatively one-dimensional, lacking comprehensive assessment. This study innovatively evaluates the clinical efficacy of elastic fixation for Lisfranc injuries through imaging, clinical indices, and plantar pressure analysis, aiming to offer more scientific and comprehensive evidence for clinical practice, validate the advantages of elastic fixation.

## Materials and methods

### General information

This study selected patients who visited our hospital from October 2022 to July 2024. Based on inclusion and exclusion criteria, 15 patients were chosen (14 male, 1 female). No patient - identifying information was accessible to the researchers during or after data collection. Their ages ranged from 25 to 46 years (mean 34.3 ± 6.6 years). Ten injuries were sports - related, and five were from traffic accidents. The mean time from injury to surgery was 6.1 ± 0.88 days (range 5–7 days). Inclusion criteria were: a visible widening (≥ 2 mm) between the second metatarsal base and medial cuneiform on weight - bearing radiographs; an MRI - confirmed Lisfranc ligament tear or a CT - confirmed simple avulsion injury; and a normal BMI (18.5 ≤ BMI < 24) without foot deformity. Exclusion criteria included: fractures elsewhere in the body or foot; open injuries; incomplete clinical data; and loss to follow - up. All methods followed the Helsinki Declaration’s guidelines. The study was approved by the Ethics Committee of the hospital, and all participants gave informed consent (Table 1).

**Table 1:** Baseline characteristics of patients

Item	Data
Gender (Male: Female, n)	14: 1
Side (Left: Right, n)	8: 7
Age($\overset-x\pm s$, years)	34.3±6.6
Type of Injury (n)	
Sports injury	10
Traffic accident injury	5
Time from Injury to Surgery ($\overset-x\pm s$,days)	6.1±0.88

**Fig. 1:**
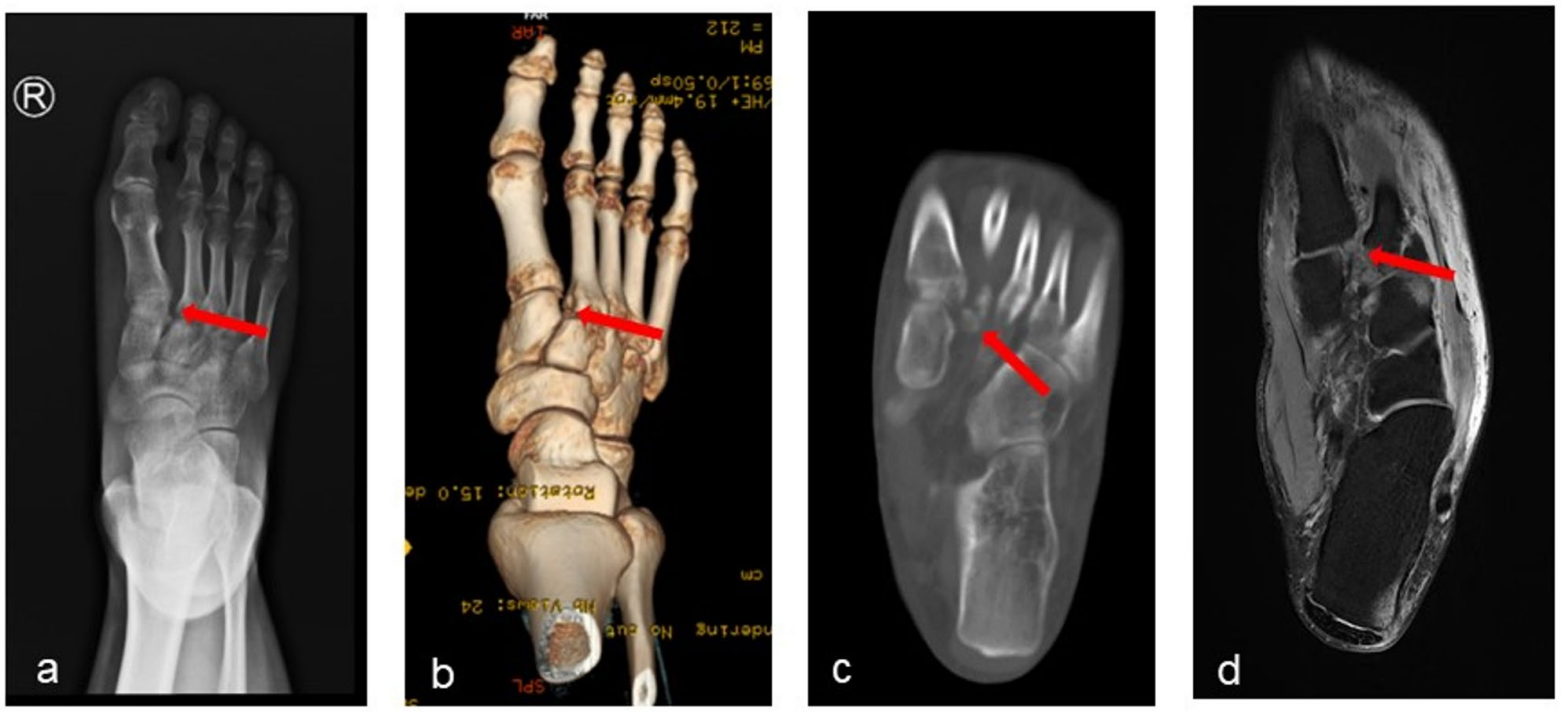
Imaging findings of Lisfranc injury : (**a**) The weight-bearing anteroposterior view of the foot shows a significantly widened gap between the second metatarsal and the medial cuneiform. **b** 、**c** CT scans reveal a significantly widened gap between the second metatarsal and the medial cuneiform, accompanied by an avulsion fracture at the base of the second metatarsal. **d** MRI shows a complete rupture of the Lisfranc ligament

### Operative procedure

**Fig. 2:**
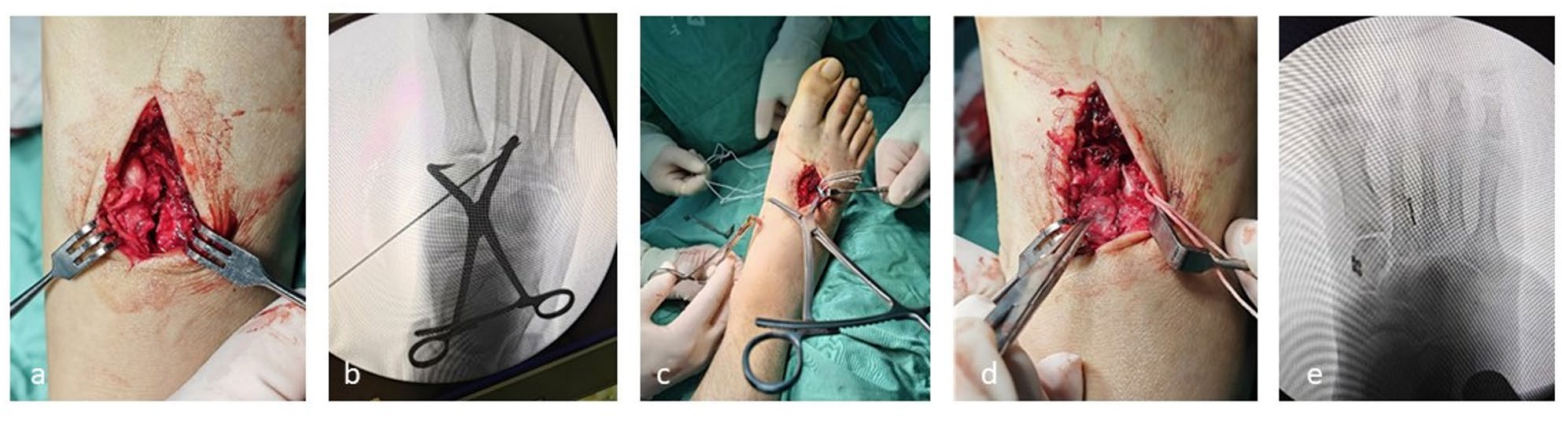
Surgical procedure (**a**) A 4 - cm longitudinal incision is made between the bases of the first and second metatarsals. **b** Under fluoroscopy, the Lisfranc joint is reduced, and the correct position of the guide pin (through the medial edge of the second metatarsal base, not the articular surface) is confirmed. **c** The elastic fixation device is installed. **d** Intraoperative examination shows good reduction and fixation. **e** Fluoroscopy is used intraoperatively to observe the reduction

Surgery was performed under combined spinal-epidural anaesthesia. After anesthesia, the patient lies supine. The surgical area is routinely disinfected, draped, and a tourniquet is applied to the upper thigh. A 4 - cm - long incision is made between the bases of the first and second metatarsals. The dorsal artery and deep peroneal nerve are protected, and the ligament injury and joint stability are assessed. The injury was stabilized using an elastic fixation system (Loop-button Titanium Plate System, Manufacturer: Double Medical Technology Co., Ltd.) during the operation. The damaged joint space is cleaned and exposed (Fig. 2a).A pointed reduction clamp is used to reduce the medial cuneiform and second metatarsal, which is then temporarily fixed with Kirschner wires. The reduction is checked by fluoroscopy (Fig. 2b༉. After satisfactory reduction, a guide pin is inserted from the medial side of the medial cuneiform to the lateral side of the second metatarsal base along the Lisfranc ligament. The position is confirmed by X - ray fluoroscopy. A hollow drill is used to enlarge the hole along the guide pin. The guide pin is removed, and a looped fixation plate is passed through the bone tunnel using a guiding pin, passing through the second metatarsal and medial cuneiform. A 1 - cm - long incision is made on the medial side of the medial cuneiform to adjust the position of the titanium plate (Fig. 2c༉. The fixation is tightened, and the pointed reduction clamp is removed. During the surgery, plantar pressure is applied to check the joint reduction. With the knee flexed 90°, a vertical load applied via the tibia: any tarsometatarsal joint widening > 2 mm or ≥ 1 mm compared with the contralateral side was deemed unstable. Or passive plantar- and dorsiflexion of the forefoot: >2 mm step-off or > 1 mm joint widening indicated instability. (Fig. 2d༉. The internal fixation position is assessed again by fluoroscopy (Fig. 2e༉. Sutures are removed after 2 weeks, and ankle joint mobility exercises begin. The brace is removed after 4 weeks, and gradual weight - bearing exercises start.

### Evaluation index

#### Radiographic assessment 

Follow-up visits were conducted at 1, 3, and 6 months postoperatively. Weight-bearing anteroposterior and lateral radiographs of the foot were taken to check for any loss of position of the tarsometatarsal joints, degenerative changes, status of internal fixation, and complications.

#### Clinical evaluation indices

The AOFAS scores and FFI were recorded preoperatively and at 1–6 months postoperatively to evaluate the surgical outcome.

#### Plantar pressure analysis

Plantar pressure data were collected and recorded preoperatively and at 1–6 months postoperatively to assess the surgical effect.

### Statistical methods

Statistical analyses were conducted with SPSS 25.0 (IBM, Chicago, IL, USA) and R 4.2.2 (R Foundation, Vienna, Austria). Quantitative data are presented as mean ± SD. A t-test was used for normally distributed data, and a Wilcoxon test for non-normally distributed data. For analyses involving more than two groups, a Friedman test (non-parametric) or ANOVA (parametric) was applied. Significance levels are marked as follows: *: *P* < 0.05, **: *P* < 0.01, ***: *P* < 0.001, ****: *P* < 0.0001, -: *P* > 0.05. Statistical significance was set at *P* < 0.05.

## Results

### Radiographic assessment

**Fig. 3:**
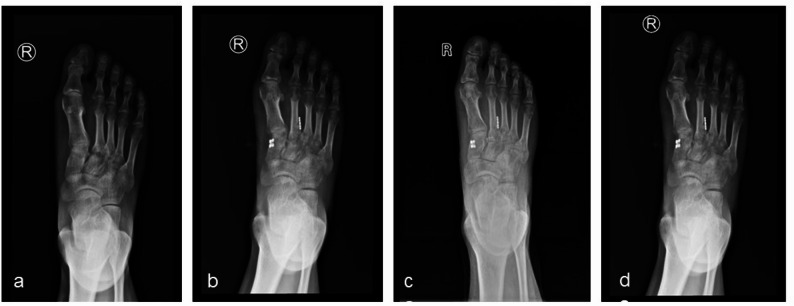
Patient A, a 36-year-old male. **a** Preoperative weight-bearing anteroposterior radiograph shows significant widening of the gap between the medial cuneiform and the second metatarsal. **b**, **c**, **d** Postoperative weight-bearing anteroposterior radiographs at 1, 3, and 6 months show good reduction of the second metatarsal, satisfactory position of internal fixation without loosening, breakage, or displacement, and no narrowing or degenerative changes in the tarsometatarsal joints

Follow-up radiographs at 1, 3, and 6 months postoperatively showed no loss of position of the tarsometatarsal joints, no degenerative changes, and symmetric joint spaces without significant narrowing or osteophyte formation. No loosening, breakage, or displacement of internal fixation devices was observed, and no complications occurred. See Fig. 3.

### Clinical evaluation indices

The AOFAS scores significantly increased from 37.6 ± 6.75 preoperatively to 92.9 ± 0.96 at six months postoperatively (*P* < 0.0001), as shown in Table 2. The FFI markedly decreased from 151.7 ± 9.31 preoperatively to 10.1 ± 2.59 at six months postoperatively (*P* < 0.0001), as shown in Table 3. Further analysis revealed that the clinical indices stabilized two months postoperatively. Significant differences were found between pre-operative values and those at post-operative month 1, but not between month 2 and subsequent time points (*P* > 0.05).

**Table 2:** Preoperative and postoperative AOFAS scores of patients

Clinical Indices	Time			Q	*P*	Multiple Comparisons
	Preop	37.6	6.75			
	1M	60.9	4.05			1M-6M >Preop****
	2M	91.4	1.60			2M-6M >1M****
AOFAS	3M	91.5	2.36	105.672	<0.001	5M >2M*
	4M	92.2	1.57			6M >2M**
	5M	92.9	1.46			6M >3M*
	6M	92.9	0.96

**Table 3:** Preoperative and postoperative FFI of patients

Clinical Indices	Time			Q	*P*	Multiple Comparisons
FFI	Preop	151.7	9.31	84.000	<0.001	
	1M	61.1	8.81			
	2M	11.9	2.58			Preop＞1M-6M ****
	3M	11.3	2.49			1M＞2M-6M****
	4M	10.7	2.15			
	5M	10.7	1.99			
	6M	10.1	2.59			

### Plantar pressure analysis

**Fig. 4:**
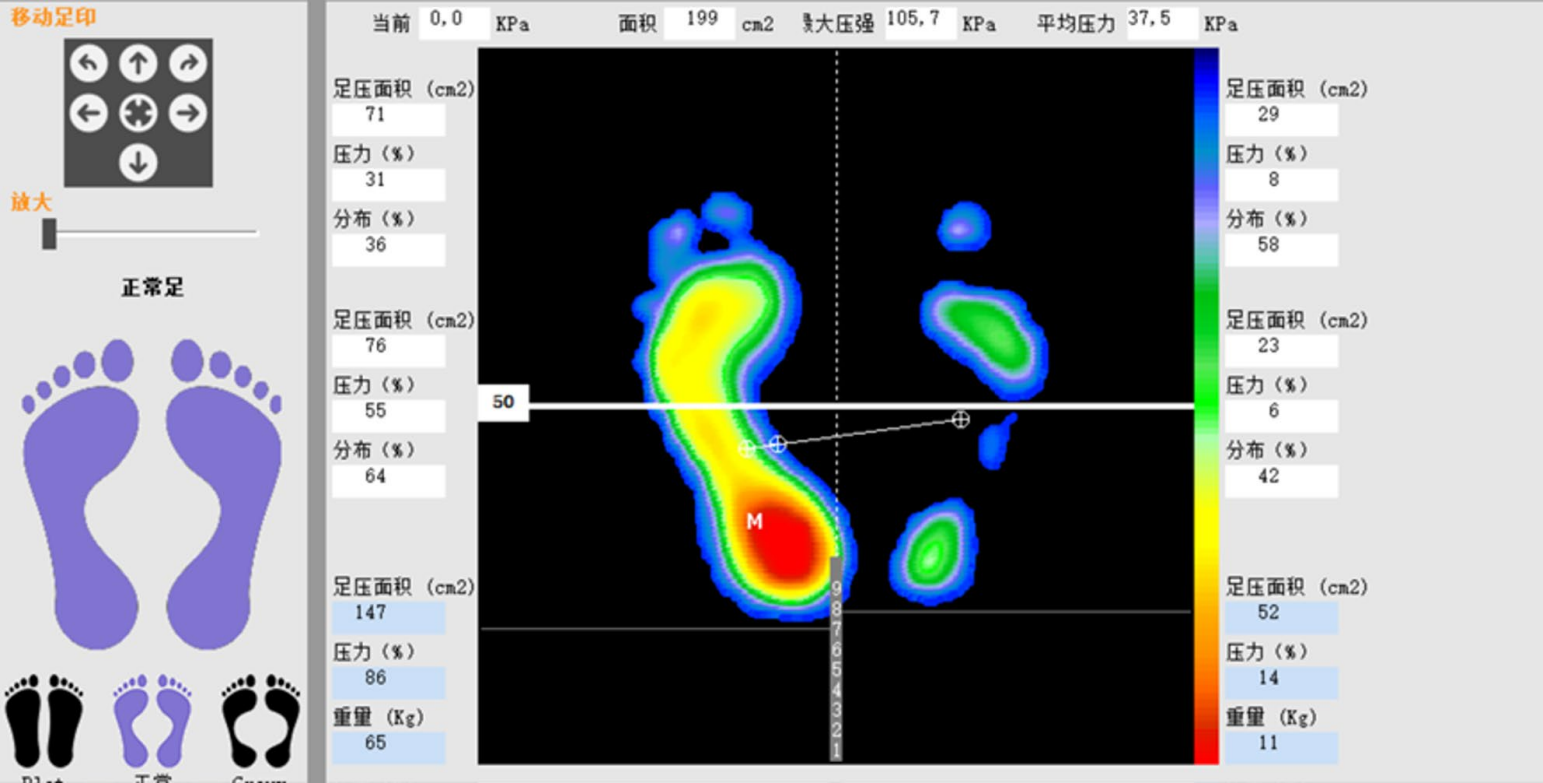
Patient Wang, post - injury plantar pressure analysis showed that the right foot (affected side) had a significantly lower pressure distribution than the healthy side. This indicates a reduction in load - bearing on the affected foot

**Fig. 5:**
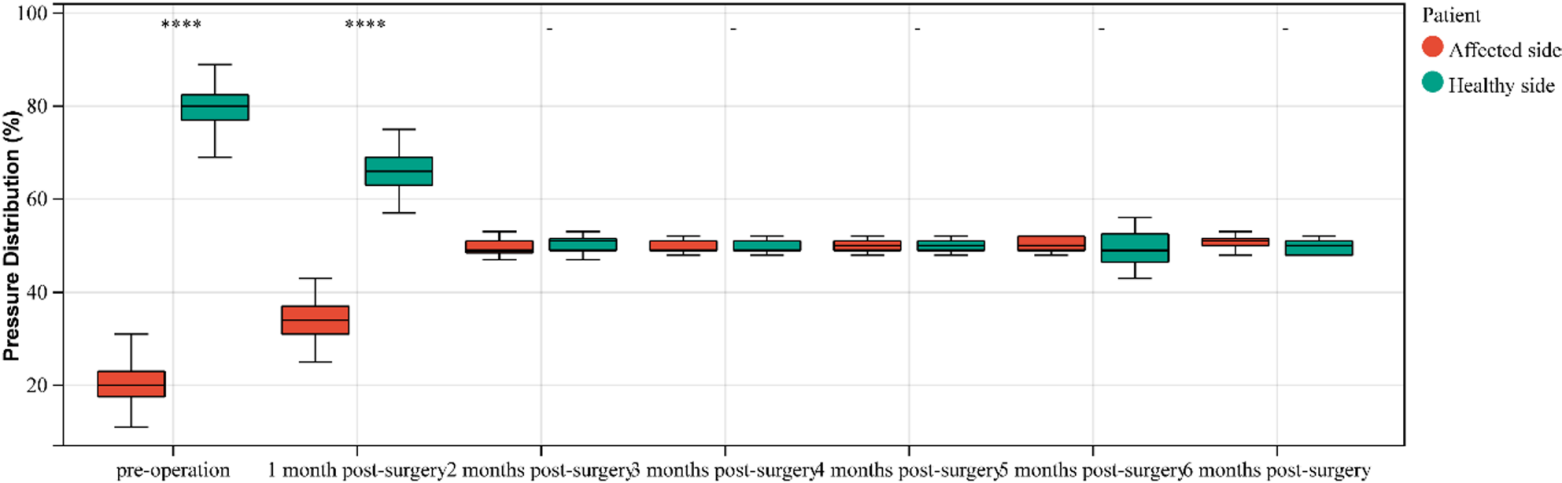
Comparison of pressure distribution between affected and healthy foot before and after surgery

Pressure distribution reflects the relative load - bearing between the affected and healthy sides (Fig. 4). The pressure distribution of the affected foot before surgery and at postoperative month 1 showed a statistically significant difference compared to postoperative month 2 (*p* < 0.05). There was no statistical difference from postoperative month 2 onwards (*p* > 0.05). See Table 4. Similarly, the pressure distribution of the healthy foot before surgery and at postoperative month 1 was significantly different from that at postoperative month 2 (*p* < 0.05). No statistical difference was found from postoperative month 2 onwards (*p* > 0.05). See Table 5. There was a statistical difference in pressure distribution between the affected and healthy feet before surgery and at postoperative month 1 (*p* < 0.05). No statistical difference was observed from postoperative month 2 onwards (*p* > 0.05). See Table 6; Fig. 5.

**Table 4:** Comparison of the pressure distribution on the affected side at different time points before and after surgery (%)

Time			Q	*P*	Multiple Comparisons
Preop	20.4	5.38			
1M	34.6	4.85			
2M	49.7	1.87			1M-6M＞Preop ****
3M	50.2	1.32	78.712	<0.001	2M-6M＞1M ****
4M	49.9	1.19			
5M	50.3	1.29			
6M	50.4	1.18

**Table 5:** Comparison of the pressure distribution on the healthy side at different time points before and after surgery (%)

Time			Q	*P*	Multiple Comparisons
Preop	79.6	5.38			
1M	65.4	4.85			
2M	50.3	1.87			Preop＞1M-6M ****
3M	49.8	1.32	72.519	<0.001	1M＞2M-6M****
4M	48.9	1.19			
5M	49.7	1.29			
6M	49.6	1.18

**Table 6:** Comparison of pressure distribution between affected and healthy foot before and after surgery (%)

Pressure Distribution (**%**)								
	Preop		1M	2M	3M	4M	5M	6M
Affected	20.4±5.38		34.6±4.85	49.7±1.87	50.2±1.32	49.9±1.19	50.3±1.29	50.4±1.18
Healthy	79.6±5.38		65.4±4.85	50.3±1.87	49.8±1.32	50.1±1.19	49.7±1.29	49.6±1.18
*P*	＜0.05		＜0.05	0.46	0.42	1.00	0.43	0.11

**Fig. 6:**
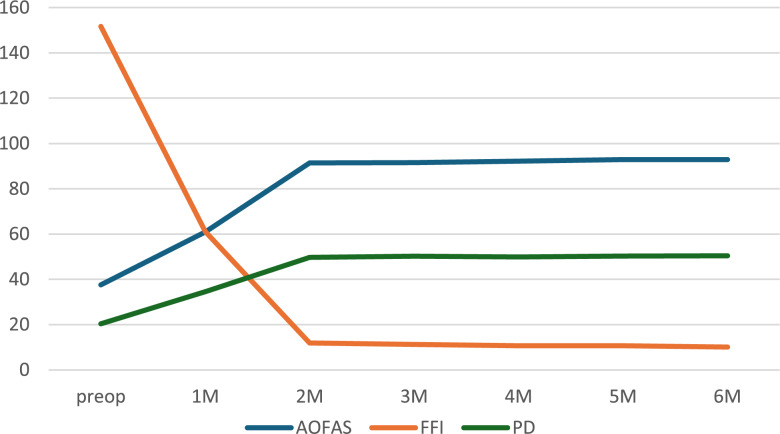
Trend plots of AOFAS, FFI, and plantar pressure distribution of the affected foot from baseline to six-month follow-up

As time progressed, the AOFAS of the affected foot rose steadily, the FFI score declined, and plantar pressure distribution improved. All values plateaued and balanced by postoperative month 2, demonstrating good functional recovery and an excellent surgical outcome. See Fig. 6.

## Discussion

Low-energy injuries, often termed occult injuries, primarily involve the Lisfranc ligament [9, 10]., and present with mild clinical symptoms. The optimal treatment for isolated Lisfranc ligament injuries remains controversial [11, 12]. While screw or plate fixation offers strong fixation, it can lead to post - traumatic arthritis and implant breakage. Animal studies indicate that absolutely stable fixation isn’t conducive to ligament healing and restoration of its biological properties [13]. In contrast, allowing controlled micromotion within a safe range applies proper tension to the healing ligament. This tension promotes the normal alignment of collagen fibers in the healing ligament, thus enhancing the overall ligament healing quality [14, 15]. Elastic fixation, which overcomes the drawbacks of rigid fixation, is gaining widespread use [16, 17]. During elastic fixation, only two traction lines are placed along the Lisfranc ligament’s anatomical path, causing less disruption to ligament healing and potentially yielding better results [18]. In this study, the clinical efficacy of elastic fixation for isolated ligamentous Lisfranc injuries was assessed through imaging, clinical evaluation indices, and plantar pressure analysis. Results show that elastic fixation offers significant benefits in function restoration, complication reduction, and rehabilitation time shortening. Imaging revealed stable reduction of the tarsometatarsal joints in all patients without degeneration or implant failure. Clinically, AOFAS scores rose from 37.6 ± 6.75 preoperatively to 92.9 ± 0.96 at six months postoperatively, FFI scores dropped from 151.7 ± 9.31 to 10.1 ± 2.59, with stabilization of clinical indices by postoperative month two. Plantar pressure analysis indicated that the affected foot’s pressure contribution increased from 20.4% preoperatively to 50.4% at six months postoperatively, with pressure distribution between both feet reaching balance by postoperative month two.

Compared with traditional screw or plate fixation, elastic fixation allows for controlled joint micromotion within a safe range, promoting ligament healing, reducing the risk of post - traumatic arthritis, and eliminating the need for secondary surgery to remove implants. Both animal experiments and clinical studies have shown that allowing a certain degree of micromotion helps improve the overall quality of ligament healing. Chona’s [8] systematic review, which included 216 patients, reported a mean postoperative AOFAS score of 90.1. D. I. Chun’s [19] study of 12 patients showed a postoperative mean AOFAS score of 93.5, consistent with this study’s postoperative 6 - month AOFAS score of 92.9 ± 0.96. Andrew D. Lachance’s [20] systematic review also indicated that patients treated with suture buttons had significantly better AOFAS at 12 - month follow - up compared with those treated with screw fixation. Elastic fixation for Lisfranc ligament reconstruction doesn’t require implant removal, has low stress, and shortens rehabilitation. Martin Sullivan [21] irst reported using elastic fixation for elite athletes’ ligamentous Lisfranc injuries. All patients, clinically and MRI-diagnosed with isolated unstable complete ligamentous Lisfranc injuries, underwent elastic fixation surgery. They were fully weight-bearing at 4 weeks postoperatively, resumed training at 9–12 weeks, and returned to full competition at 12–16 weeks. Researchers noted that larger suture button devices can significantly shorten recovery time. Cho [22] retrospectively compared 164 consecutive Lisfranc injury patients, with 63 included and divided into two groups: 32 with traditional screw fixation and 31 with suture button fixation. The study measured clinical and radiological results preoperatively, at 6 months, 1 year, and final follow-up, along with plantar pressure at 6 months postoperatively, and assessed postoperative complications. Results showed that at 6 months postoperatively, the suture button group had better AOFAS scores than the screw fixation group. Patients in this study were fully weight-bearing at 12 weeks postoperatively, while Delman C [23], Jain K [24] and Tzatzairis T [25] reported some patients being fully weight-bearing at 6 weeks. In our follow-up, some patients achieved full weight-bearing within 4–6 weeks. Jain K used elastic fixation in athletes, who returned to competition at 21 weeks postoperatively, as did Charlton [26] believed that larger suture buttons enabled earlier weight-bearing and return to competition within 12–16 weeks. We used 5 mm diameter button plates, which might explain our patients’ early weight-bearing. Meloria Hoskins [27] treated nine patients with ligamentous Lisfranc injuries using the InternalBrace device for elastic fixation. Postoperatively, full weight-bearing was achieved at an average of 6.8 weeks, and return to work/sport occurred at 13.2 weeks—comparable to Tight-rope literature and faster than screw meta-analyses (19–22 weeks). There were no infections, no hardware failures, and no loss of reduction, indicating favorable short-term outcomes. Although the specific elastic fixation devices used were different and the number of patients followed was small, the results still demonstrate that elastic fixation is effective and significantly shortens rehabilitation time.

Consistent with other studies, our patient group had no postoperative issues like wound problems, device loosening, implant failure, recurrent instability, or loss of reduction. Unlike traditional screw or plate fixation, elastic fixation eliminates the need for second surgery to remove implants, significantly shortening recovery time. Mora [28] studied 33 amateur athletes and found all underwent implant removal within 6 months and returned to unrestricted sports at 8 months. Deol [29] reported on the effectiveness of traditional screw fixation for elite athletes’ Lisfranc injuries, with an average return to training time of 19.3 weeks and return to competition time of 24.1 weeks due to the need for secondary screw removal. Nevertheless, the pitfalls reported by other investigators should be kept in mind. Koroneos ZA [16] documented loss of reduction in some patients after elastic fixation; Jain K [24] observed dorsal foot pain that resolved only after device removal; and Chona [24] described button-to-bone cut-out that necessitated revision surgery in a subset of cases. Although these complications were not observed in our limited cohort, they remain potential risks.

In healthy adults during standing or walking, weight - bearing mainly focuses on the hindfoot and forefoot [30], This distribution maintains balance and disperses pressure, reducing the risk of injury from prolonged high pressure on a single area.Simultaneously, the pressure distribution between both feet is basically symmetrical [31]. This symmetry ensures body stability during activities and prevents issues like postural instability or muscle tension [32]。This study analyzed plantar pressure preoperatively and at 1–6 months postoperatively, revealing patients’ load adaptation during rehabilitation. The affected foot’s pressure distribution increased from 20.4% preoperatively to 50.4% at six months postoperatively, indicating it bore more load over time. In the early postoperative period, especially the first month, the pressure distribution saw a significant increase, showing a pronounced shift from the healthy to the affected foot. Conversely, pressure on the healthy foot decreased from 79.6% preoperatively to 49.6% at six months postoperatively, reflecting a gradual reduction in its load - bearing burden. These changes in pressure distribution highlight the process of load transfer, showing that the affected foot bears more weight over time, while the healthy foot’s burden lessens. This balanced load distribution by two months postoperatively indicates that patients with elastic fixation recover quickly and can resume weight - bearing activities early.

Nevertheless, this study has certain limitations and the findings are exploratory and hypothesis-generating rather than definitive. The sample size was small and the follow-up period was short. Given the small sample size and short follow-up period, large-scale, multicenter studies incorporating dynamic gait-cycle loading and long-term follow-up are still needed to further validate the safety and efficacy of elastic fixation across diverse populations and loading conditions. It should be emphasized that our cohort was restricted to patients with MRI- or CT-confirmed isolated ligamentous injuries, excluding those with fractures, open injuries, or chronic instability. Therefore, our conclusions cannot be extrapolated to all Lisfranc injury patterns. Furthermore, the exclusion of overweight patients and complex fractures further limits the generalizability of the findings to real-world clinical practice. Future research should further evaluate the clinical efficacy of combining elastic fixation with other internal fixation techniques in complex Lisfranc injuries characterized by concurrent ligamentous disruption and fracture-dislocation, thereby broadening the indications for elastic fixation.

## Conclusion

In this retrospective case series of carefully selected patients with isolated ligamentous Lisfranc injuries (isolated injury only, no open wounds, normal BMI, no foot deformity), elastic fixation produced rapid functional improvement, restored plantar-pressure balance by 2 months and allowed early weight-bearing without early complications. However, owing to the small sample and short follow-up, the results must be interpreted as preliminary. Multicentre trials with larger samples, longer follow-up, and more diverse populations are required to confirm the indications, long-term outcomes, and potential complications of elastic fixation before its widespread adoption.

## Data Availability

The datasets used and/or analyzed during the current study are available from the corresponding author on reasonable request.

## Abbreviations

AOFAS: American Orthopaedic Foot and Ankle Society
FFI: Foot Function Index
